# Dietary Correlates of Oral and Gut Microbiota in the Water Monitor Lizard, *Varanus salvator* (Laurenti, 1768)

**DOI:** 10.3389/fmicb.2021.771527

**Published:** 2022-01-06

**Authors:** Yu Du, Jun-Qiong Chen, Qian Liu, Jian-Chao Fu, Chi-Xian Lin, Long-Hui Lin, Hong Li, Yan-Fu Qu, Xiang Ji

**Affiliations:** ^1^Jiangsu Key Laboratory for Biodiversity and Biotechnology, College of Life Sciences, Nanjing Normal University, Nanjing, China; ^2^Hainan Key Laboratory of Herpetological Research, College of Fisheries and Life Science, Hainan Tropical Ocean University, Sanya, China; ^3^MOE Key Laboratory of Utilization and Conservation for Tropical Marine Bioresources, Hainan Tropical Ocean University, Sanya, China; ^4^Hangzhou Key Laboratory for Ecosystem Protection and Restoration, College of Life and Environmental Sciences, Hangzhou Normal University, Hangzhou, China; ^5^Zhejiang Provincial Key Laboratory for Water Environment and Marine Biological Resources Protection, College of Life and Environmental Sciences, Wenzhou University, Wenzhou, China

**Keywords:** 16S rRNA gene sequencing, food, gut microbiota, oral microbiota, *Varanus salvator*, amplicon sequence variants

## Abstract

Numerous studies have demonstrated that food shapes the structure and composition of the host’s oral and gut microbiota. The disorder of oral and gut microbiota may trigger various host diseases. Here, we collected oral and gut samples from wild water monitor lizards (*Varanus salvator*) and their captive conspecifics fed with bullfrogs, eggs, and depilated chicken, aiming to examine dietary correlates of oral and gut microbiota. We used the 16S rRNA gene sequencing technology to analyze the composition of the microbiota. Proteobacteria and Bacteroidota were the dominant phyla in the oral microbiota, and so were in the gut microbiota. The alpha diversity of microbiota was significantly higher in the gut than in the oral cavity, and the alpha diversity of oral microbiota was higher in captive lizards than in wild conspecifics. Comparing the relative abundance of oral and gut bacteria and their gene functions, differences among different animal groups presumably resulted from human contact in artificial breeding environments and complex food processing. Differences in gene function might be related to the absolute number and/or the taxonomic abundance of oral and gut microorganisms in the wild and the water environment. This study provides not only basic information about the oral and gut microbiota of captive and wild water monitor lizards, but also an inference that feeding on frogs and aquatic products and reducing human exposure help water monitor lizards maintain a microbiota similar to that in the wild environment.

## Introduction

Microbes affect many aspects of their host, including its life history (Videvall et al., 2019), immune regulation (Grice and Segre, 2011), healthy status (Alcaraz et al., 2012), and adaptability (Zhang et al., 2018b). The host has plenty of space for microbial colonization, including the body surface (Timm et al., 2020), oral cavity (Lu et al., 2019), and intestinal tract (Kartzinel et al., 2019). Microbial genomes from the gut encode more than 3.3 million genes, which are 10–100 times larger than their hosts (Qin et al., 2010). Species diversity and richness of host microbiomes can be very high. For example, there are approximately 700 kinds of microorganisms in the human oral cavity (Lamont et al., 2018). With the development of sequencing technology, increasingly more attention has been paid to the evolution and functional role of host microorganisms (Lamont et al., 2018; Lavrinienko et al., 2018; Lu et al., 2019).

The oral cavity consists of a complex system of tissues and organs that provide a highly heterogeneous habitat for microorganisms, of which bacteria are the main group (Kilian, 2018; Lu et al., 2019). The dominant phyla of human oral bacteria are Firmicutes, Bacteroidetes, Proteobacteria, and Actinobacteria (Welch et al., 2016). The oral microbiota is an important part of the host microbiota. In human beings, oral microbial dysbiosis leads not only to oral diseases, such as periodontal disease (Ohlrich et al., 2010) and oral cancer (Yang et al., 2018), but also to systemic diseases such as rheumatoid arthritis (Graves et al., 2019), diabetes (Ohlrich et al., 2010), and digestive diseases (Ray, 2017). Ontogenetic shifts in oral microbiota have been reported for wild organisms, such as the Cooper’s hawk *Accipiter cooperii* (Taylor et al., 2019), and habitat-related variation in the oral microbiome has been detected in the Komodo dragon *Varanus komodoensis* (Hyde et al., 2016).

The gut microbiota also affects many aspects of host life, including growth (Pennisi, 2016), behavior (Proctor et al., 2017), metabolism (Kartzinel et al., 2019), reproduction (Leftwich et al., 2017), and inflammatory/immune responses (Montalban-Arques et al., 2015). Gut microbial dysbiosis may trigger chronic inflammatory diseases and certain types of cancer (Lalles, 2016; Tsvetikova and Koshel, 2020). The coevolution between the host and its gut microbes has been studied in a broader range of animal taxa. Previous studies generally show that the composition of gut microbiota is taxa-specific. As for the tetrapod gut microbiota, Firmicutes and Bacteroidetes are the dominant bacterial phyla in amphibians (Vences et al., 2016), reptiles (Qin et al., 2019), and mammals (Kartzinel et al., 2019), and Proteobacteria and Firmicutes are the dominant bacterial phyla in birds (Dewar et al., 2014).

The composition of gut microbes is influenced by many factors, such as diet (Tang et al., 2020), habitat environment (Zhang et al., 2018b), health status (Zeevi et al., 2019), age (Scott et al., 2017), gender (Mueller et al., 2006), and phylogenetic relationship (Kartzinel et al., 2019). For example, captivity affects diversity, abundance, and functional pathways of gut microbiota in the northern grass lizard *Takydromus septentrionalis* (Zhou et al., 2020), and so does altitude in the Qinghai toad-headed lizard *Phrynocephalus vlangalii* (Zhang et al., 2018b). The composition of oral microbes is also affected by multiple factors and displays ontogenetic shifts (Lu et al., 2019). In *A. cooperii*, for example, age is an important determinant of the oral microbiota (Taylor et al., 2019). Oral microbiota can colonize in the gut in some ways (Qin et al., 2014; Abed et al., 2016). There is evidence in humans with liver cirrhosis that the oral commensals invade the gut (Qin et al., 2014). However, it is worth noting that the relationship between the oral and gut microbiota is not consistent among different species. For example, the oral microbiota is associated with the gut microbiota in human children (Liu et al., 2021), but much less so in the great tit *Parus major* (Kropáčková et al., 2017).

Reptiles are an important group of terrestrial vertebrates, but their oral and gut microbiota remain poorly known, especially when compared with other vertebrate taxa (Colston and Jackson, 2016). Inputting gut microbiota and vertebrate group name as our article title, abstract, and keywords in the Scopus database, we found 3,391 reports or articles, 1,471 for mammals, 575 for birds, 34 reptiles, 46 for amphibians, and 1,265 for fish. Replacing gut microbiota with oral microbiota, we found 331 reports or articles, 194 for mammals, 56 for birds, nine for reptiles, four for amphibians, and 68 for fish. Previous studies on lizards are limited to a few species of the genera *Diplolaemus*, *Eublepharis*, *Phrynocephalus*, *Shinisaurus*, *Takydromus*, and *Varanus* (Ibarguengoytía et al., 2005; Zancolli et al., 2015; Zhang et al., 2018b; Tang et al., 2020; Zhou et al., 2020; Soopramanien et al., 2021). Here, we used high-throughput sequencing to study dietary correlates of oral and gut microbiota in the water monitor lizard, *Varanus salvator* (Laurenti, 1768). This large-sized (up to 1,170 mm snout-vent length; Traeholt, 1998) oviparous lizard is listed in CITES Appendix II and has a range covering Bangladesh, Brunei, Indo-China Peninsula, Indonesia, Northeast India, South-Southwest China, and Sri Lanka (Du et al., 2014). We address two main questions: (1) Does food affect the oral and gut microbiota? and (2) Is there any relationship between oral and gut microorganisms?

## Materials and Methods

### Sample Collection

We used 25 adult lizards without any signs of disease (including ectoparasites) to conduct this study. Of these lizards, 22 (8♀♀14♂♂, hereafter captive lizards) were from a captive population established 13 years ago by 18 confiscated illegally traded adults at Hainan Tropical Ocean University, and three (2♀♀1♂, hereafter wild lizards) from a natural population in Jiaxi, Hainan, China. Captive lizards were at 4–5 years old, and wild lizards were at unknown ages. We randomly divided captive lizards into three groups and then kept them individually in 3 × 2.8 × 2 (length × width × height) m indoor enclosures, where seven (2♀♀5♂♂, hereafter egg-fed lizards) were fed with eggs (*Gallus gallus*), seven (2♀♀5♂♂, hereafter frog-fed lizards) with captive-raised bullfrogs (*Rana catesbiana*), and eight (4♀♀4♂♂, hereafter chicken-fed lizards) with depilated chicken (*G. gallus*). We fed captive lizards with sufficient food (sterilized with UV lamp for 1 h in advance) for 1 h at 3-day intervals. No mating behavior was observed during the experiment. Lizards of four different groups (three groups of captive lizards and one group of wild lizards) did not differ from each other in mean values for snout-vent length (*F*_3,20_ = 0.911, *p* = 0.453) and body mass (*F*_3,20_ = 1.550, *p* = 0.233). We used cotton swabs to collect oral and gut (cloacal) microbial samples from each lizard in late September 2020, 2 months after captive lizards were moved into the three enclosures. When collecting samples, we first rinsed a lizard’s mouth or cloaca with sterile water and then scraped the mouth (along the lining of the mouth and gums) or cloaca (always 70 mm depth) using a cotton swab. We placed each cotton swab with adhering microbial sample in a sterile 50 ml conical tube with 10–20 ml normal saline solution, numbered each tube with a pencil, and sealed it with parafilm wrap. All tubes were stored at −20°C for later use. Our experimental procedures complied with current laws on animal welfare and research in China, and were approved by the Animal Research Ethics Committee of Nanjing Normal University (Permit no. IACUC 20200511).

### DNA Extraction, PCR Amplification, and Sequencing of Samples

We used the HiPure Stool DNA Kits (Magen, Guangzhou, China) to extract the microbial DNA from the swabbed fecal samples according to the manufacturer protocols, the ultraviolet spectrophotometer to measure its concentration and purity, and 0.8% agarose gel electrophoresis at 120 V for 20 min to detect its integrity. The V3–V4 region of the 16S rRNA gene was amplified by PCR using 341F (5′-CCTACGGGNGGCWGCAG-3′) and 806R (5′-GGACTACHVGGGTATCTAAT-3′). PCR was performed in the 50 μl reaction system consisting of 10 μl Q5 reaction buffer (5×), 10 μl Q5 High GC enhancer (5×), 1.5 μl dNTPs (2.5 mM), 1.5 μl of each primer (10 μM), 0.2 μl Q5 High-Fidelity DNA Polymerase (5 U/μl), and 50 ng of template DNA. Related PCR reagents were from New England Biolabs, United States. The PCR thermal cycling conditions were as follows: initial denaturation at 95°C for 5 min, followed by 30 cycles of denaturation at 95°C for 1 min, at 60°C for 1 min, and at 72°C for 1 min, and a final extension at 72°C for 7 min. The PCR products were visualized using the 2.0% agarose gel, purified by the AxyPrep DNA Gel Extraction Kit (Axygen Biosciences, Union City, CA, United States) according to the manufacturer’s instructions and quantified using ABI StepOnePlus Real-Time PCR System (Life Technologies, Foster City, United States). Purified amplicons DNA libraries were pooled in equimolar and paired-end sequenced (PE250) on an Illumina platform (Nova-seq 6000) in accordance with the manufacturer’s protocols.

### Quality Control and Data Standardization

We carried out quality control to optimize the quality of raw data. Data processing was conducted using Quantitative Insights into Microbial Ecology 2 (QIIME2; Bolyen et al., 2019). Paired-end sequences were imported into QIIME2. We used the DADA2 package to filter and truncate low-quality reads, including those containing unknown nucleotides and primer sequences (Callahan et al., 2016). The filtered reads were denoised using DADA2 to obtain paired-end reads, and these reads were merged as raw amplicon sequence variants (ASV) with a minimum overlap of 12 bp. Then, the UCHIME algorithm was used to identify and delete chimera sequences and obtain clean ASV sequences (Edgar et al., 2011). These sequences were then submitted to the National Genomics Data Center (NGDC) GSA database (accession number CRA004563).

The RDP Classifier 2.2 was used to classify ASVs into organisms by a naive Bayesian model based on the Silva 138 Database at the confidence threshold of 99% (Quast et al., 2012). We used the QIIME2 to calculated the sequencing depth index and thereby evaluate the adequacy of sequencing data. Then, a sequencing depth for each sample was visualized using R 3.6 (R Development Core Team, 2020). The ASVs abundance information was standardized using the sample with the least sequence number for further analysis. We retained ASVs with the number of ASVs greater than 10 in at least two samples for further analysis to avoid large partial sample deviations.

### Estimation of Alpha and Beta Diversity

To analyze alpha diversity, we used QIIME2 to calculate the community richness (Chao1 index), community diversity (Shannon diversity index), and community evenness (Pielou’s evenness index) for each microbial sample. These indicators were then visualized by the R software platform and represented the community richness, evenness, diversity, and coverage of oral and cloacal microbiota. We used one-way ANOVA to test differences in alpha diversity in the oral and gut microbiota among animal groups [(egg-, frog-, and chicken-fed) captive lizards and wild lizards], and paired-sample *t*-test to test differences between sampling sites (oral cavity and gut). We used G*Power 3.1 to calculate power for paired-sample *t*-test and one-way ANOVA (Faul et al., 2009). G*Power analysis detected more than 99% power for paired-sample *t*-test (between sampling sites) and more than 95% power for one-way ANOVA (among animal groups).

For the beta diversity, we used principal coordinates analysis (PCoA) and analysis of similarity (ANOSIM) to show the differences in community structure of oral and gut microbiota among different animal groups. PCoA based on the ASV level was conducted to reveal the differences among groups on a two-dimensional graph, and ANOSIM based on the bray_curtis distance with 999 permutations was conducted to assess the statistical significance and proportion of variance explained by the contrast in microbial composition between oral and gut microbiota. ANOSIM based on permutation test is superior to parametric test for small sample data and statistically more powerful than Kruskal-Wallis test. Our data achieved high power in this study, so ANOSIM is suitable for current data analysis. Individual identity was considered as a constraint for permutations in ANOSIM models to avoid the nondependent effects. Then, ANOSIM was used to compare the differences among animal groups for oral and gut microbiota, respectively. We used the Mantel test to test the relationship between the oral and gut microbiota. We used the linear discriminant analysis effect size (LEfSe; Segata et al., 2011) to compare the microbial abundances from the phylum to family levels among animal groups and thereby determined the effects of diet on oral and gut microbiota. In addition, the linear discriminatory analysis (LDA) was conducted to evaluate the effect size for each selected classification. Only the bacterial taxa with a log LDA score >4 (over 4 orders of magnitude) were used in this analysis. We used Mann-Whitney *U* test to verify whether the bacteria detected by LDA had a higher relative abundance of the oral or gut bacteria. We used kwpower function in R package MultNonParam to calculate power for LEfSe because LEfSe approach rests on a Kruskal-Wallis test (Kolassa and Jankowski, 2021). Our analyses had 84% power to detect differences in the bacterial relative abundances among animal groups.

### Gene Function Predication

PICRUSt was used to explore the functional differences among the bacterial communities based on the Kyoto Encyclopedia of Genes and Genomes (KEGG) database (Kanehisa, 2019). These gene functions were then classified and allocated to the corresponding KEGG pathways (Langille et al., 2013). Gene functions are stored in the KEGG Orthology (KO) database, where each KO is defined as a functional homology of genes and proteins. Higher-level functions are represented by networks of molecular interactions, reactions, and relationships in the form of KEGG pathway diagrams (Kanehisa, 2019). The numbers of functional genes in each pathway were counted to assess their relative abundances in different groups. We used LEfSe to compare the differences in the relative abundances of predicated functions among animal groups, and LDA to determine the effect size for each selected functional category. Only the gene functional category with a log LDA score >3 (over 3 orders of magnitude) was used in this analysis. The result of power analysis is consistent with the result of the relative abundances of microbiota, indicating that our statistical method is feasible. All values were presented as mean ± SE, and the significance level was set at *α* = 0.05.

## Results

### Oral and Gut Bacterial Sequencing and ASVs Classification

We obtained 2,322,679 oral and 2,528,067 gut raw reads from the 25 lizards. After quality control, we obtained 1,568,878 oral and 1,618,831 gut high-quality reads, with an average of 62,755 oral and 64,753 gut reads per sample (Supplementary Table 1). As observed from the rarefaction curves based on ASVs, the assessed values regarding the richness of oral and gut bacterial communities were stable and unbiased for each sample (Supplementary Figure S1). We identified 1,354 ASVs in both oral and gut microbiota, with 1,008 in the oral microbiota, 1,182 in the gut microbiota, and 96–366 ASVs in each sample (Supplementary Table 2). Specifically, these 1,354 ASVs of oral and gut microbiota could be allocated to 23 phyla, 47 classes, 111 orders, 204 families, and 408 genera based on phylogenetic classification for the 50 microbial samples.

### Composition and Abundance of Microbiota in the Oral Cavity and Gut

**Figure 1:**
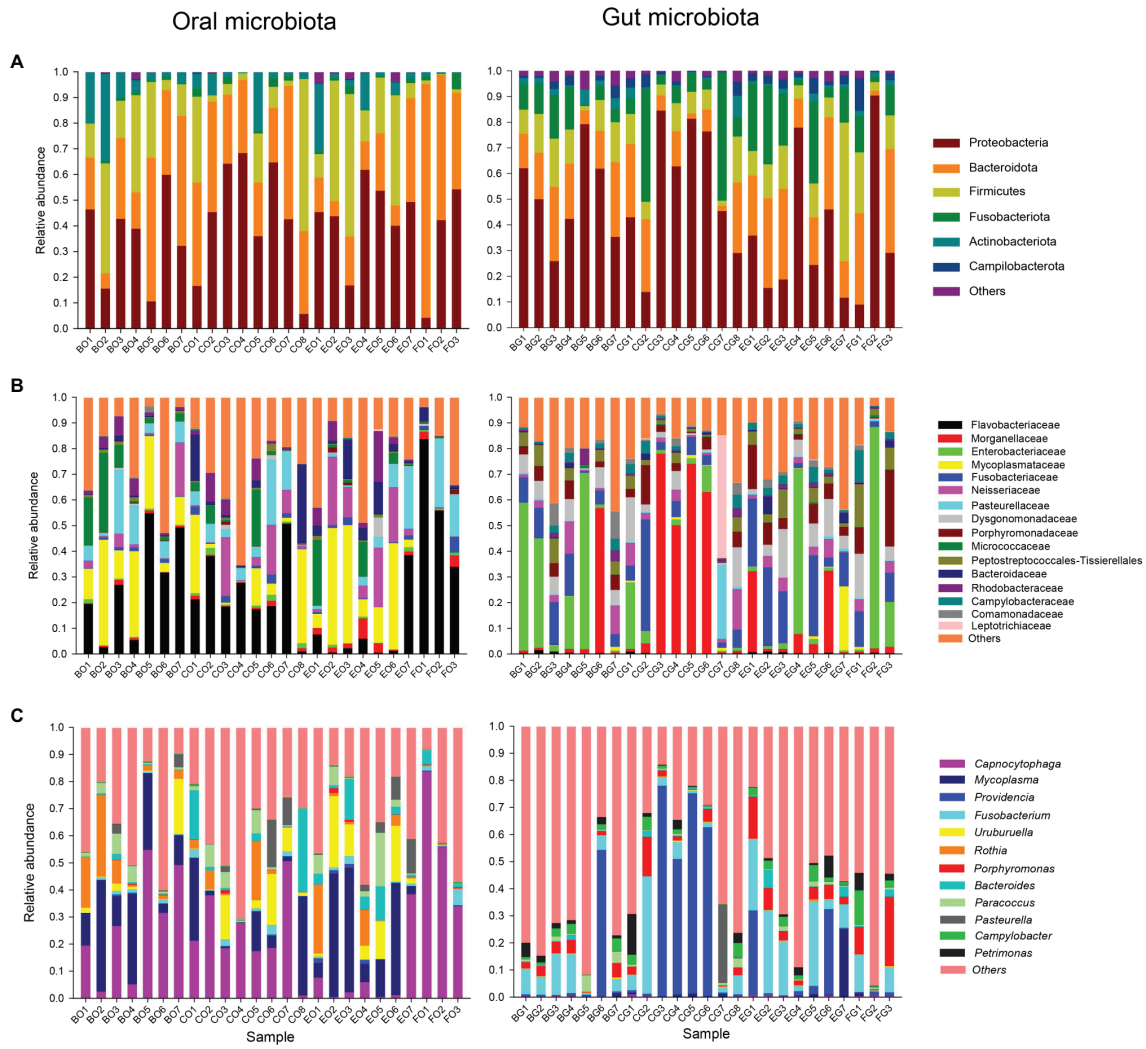
The relative abundance of the oral and gut microbiota in each group at the phylum **(A)**, family **(B)**, and genus **(C)** levels. Each color in a plot represents a taxonomic group, of which the name is shown on the right side of the plot. The color for “others” indicates all other phyla **(A)**, families **(B)**, or genera **(C)** combined, of which the names are not listed in each plot. The first letter in each group indicates the item of food, such as B for bullfrogs, C for depilated chicken, E for eggs, and F for wild prey. The second letter in each group indicates a type of microbiota, G for the gut microbiota, and O for the oral microbiota.

The top four dominant phyla were Proteobacteria (40.1 ± 3.7%), Bacteroidota (31.3 ± 3.9%), Firmicutes (19.3 ± 3.7%), and Actinobacteriota (7.1 ± 1.8%) in the oral microbiota, and Proteobacteria (46.1 ± 5.0%), Bacteroidota (20.0 ± 2.4%), Fusobacteriota (13.9 ± 2.6%), and Firmicutes (12.6 ± 2.0%) in the gut microbiota (Figure 1A). The dominant families with a relative abundance >3% were Flavobacteriaceae (24.6 ± 4.3%), Mycoplasmataceae (15.7 ± 3.2%), Pasteurellaceae (8.6 ± 1.7%), Neisseriaceae (7.3 ± 1.8%), Micrococcaceae (5.9 ± 1.8%), Bacteroidaceae (4.3 ± 1.4%), and Rhodobacteraceae (3.7 ± 0.9%) in the oral microbiota, and Enterobacteriaceae (16.8 ± 5.1%), Morganellaceae (16.7 ± 5.1%), Fusobacteriaceae (11.9 ± 2.2%), Dysgonomonadaceae (7.1 ± 1.1%), Porphyromonadaceae (6.2 ± 1.3%), Peptostreptococcales-Tissierellales (4.7 ± 0.8%), and Neisseriaceae (4.1 ± 0.8%) in the gut microbiota (Figure 1B). The dominant genera with a relative abundance >3% were *Capnocytophaga* (24.5 ± 4.3%), *Mycoplasma* (15.7 ± 3.2%), *Uruburuella* (6.3 ± 1.6%), *Rothia* (5.9 ± 1.7%), *Bacteroides* (4.3 ± 1.4%), and *Paracoccus* (3.4 ± 0.9%) in the oral microbiota, and *Fusobacterium* (10.8 ± 2.2%) in the gut microbiota (Figure 1C).

### Dietary Correlates of Oral and Gut Microbiota

**Figure 2:**
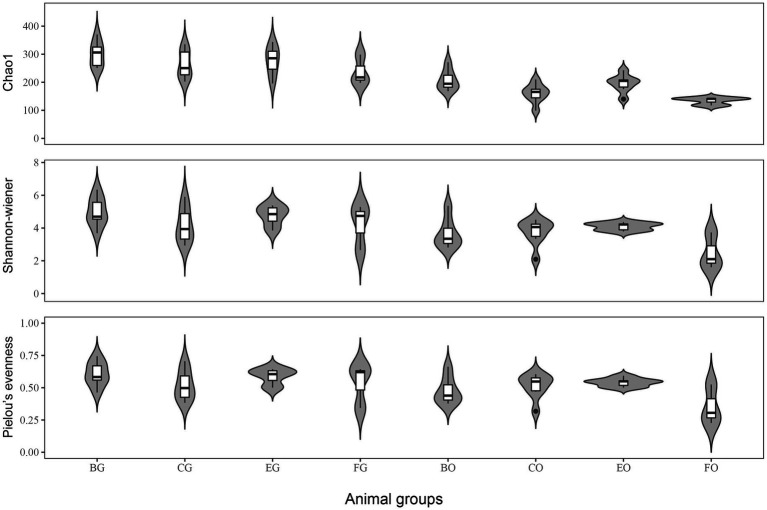
The alpha diversity indexes of gut microbiota among the four animal groups, including Chao1, Shannon-Weiner and Pielou’s evenness. See Figure 1 for the definition of every group.

All diversity indexes were greater in the gut microbiota than in the oral microbiota (all *t* > 2.73, *df* = 24 and all *p* < 0.01). One-way ANOVA showed that Chao1 index (*F*_3,21_ = 5.18, *p* < 0.01), Shannon (*F*_3,21_ = 3.18, *p* < 0.05), and Pielou’s evenness (*F*_3,21_ = 3.26, *p* < 0.04) indexes differed among lizard groups, being significantly greater in frog- (for Chao1 index) and egg-fed (for Shannon and Pielou’s evenness indexes) captive lizards than in wild lizards (Figure 2). For the gut microbiota, all diversity indexes did not differ significantly among lizard groups (Figure 2; all *F*_3,21_ < 1.33 and all *p* > 0.29).

**Figure 3:**
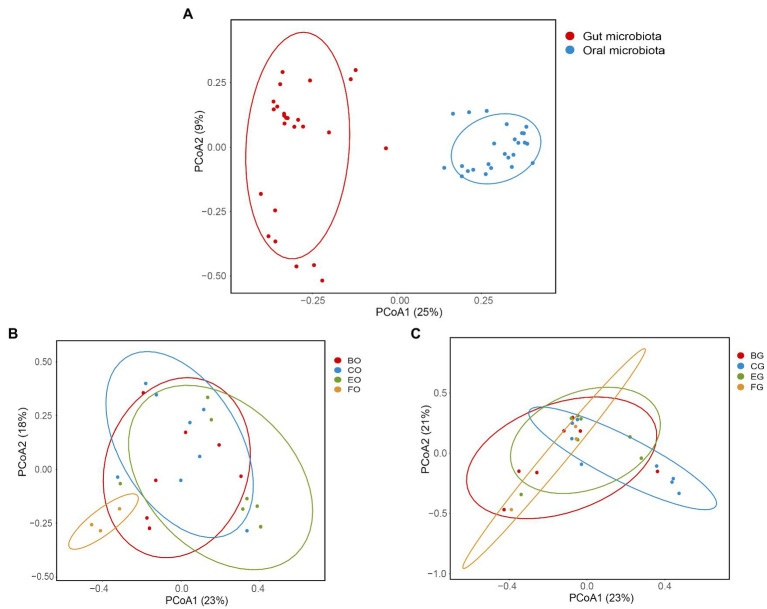
Oral and gut microbial diversity in the eight groups **(A)**, and the oral **(B)** and gut **(C)** microbial diversity among animal groups. Principal coordinates analysis of Bray-Curtis distance matrix for bacterial community diversity. See Figure 1 for the definition of every group.

Positions of the bacterial composition on a two-dimensional plane defined by the first two axes of PCoA differed between the oral cavity and gut (Figure 3A; ANOSIM: *r* = 0.94, *F*_1,48_ = 14.93, *p* < 0.001). The Mantel test showed no significant correlation between the oral and gut microbiota (*r* = −0.04, *p* = 0.69). Positions of the oral (Figure 3B; ANOSIM: *R* = 0.24, *F*_1,23_ = 2.10, *p* = 0.005) rather than the gut (Figure 3C; ANOSIM: *R* = 0.10, *F*_1,23_ = 1.42, *p* = 0.08) bacterial composition on a two-dimensional plane defined by the first two axes of PCoA differed among animal groups.

**Figure 4:**
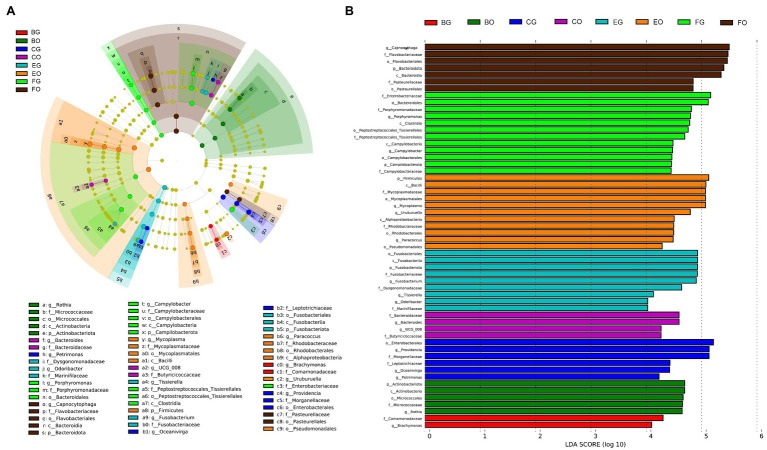
Differences in oral and gut bacterial taxa among the eight groups are determined by linear discriminant analysis effect size (LEfSe; **A**). Linear discriminatory analysis (LDA) scores reflect the differences in relative abundance among the eight groups **(B)**. See Figure 1 for the definition of every group. The letters “o,” “f,” and “g” indicate order, family, and genus, respectively.

**Figure 5:**
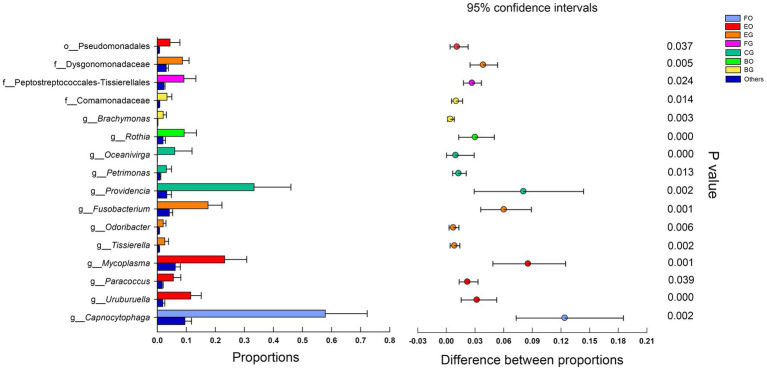
Microbial relative abundance differences in different groups. The error bars are standard deviations error. The star indicates (*p* < 0.05) using Mann-Whitney *U* test. See Figure 1 for the definition of every group. The letters “o,” “f,” and “g” indicate order, family, and genus, respectively.

LEfSe analysis revealed that cloacal bacteria of the families Comamonadaceae (*LDA* = 4.21, *p* = 0.0008) in frog-fed lizards, Dysgonomonadaceae (*LDA* = 4.64, *p* = 0.0003) in egg-fed lizards, Enterobacteriaceae (*LDA* = 5.16, *p* = 0.002), and Peptostreptococcales-Tissierellales (*LDA* = 4.69, *p* = 0.0001) in wild lizards displayed a higher relative abundance (Figure 4). The genera with a higher proportion in the gut microbiota were *Providencia* (*LDA* = 5.13, *p* = 0.0009), *Oceanivirga* (*LDA* = 4.42, *p* < 0.0001), and *Petrimonas* (*LDA* = 4.23, *p* < 0.0001) in chicken-fed lizards, *Faecalitalea* (*LDA* = 4.07, *p* = 0.05), *Fusobacterium* (*LDA* = 4.90, *p* = 0.0008), *Tissierella* (*LDA* = 4.13, *p* < 0.0001), and *Odoribacter* (*LDA* = 4.03, *p* = 0.0004) in egg-fed lizards, and *Porphyromonas* (*LDA* = 4.80, *p* < 0.0001) and *Campylobacter* (*LDA* = 4.47, *p* < 0.0001) in wild lizards (Figure 4). In the oral microbiota, Pseudomonadales (*LDA* = 4.25, *p* = 0.045) at the order level in egg-fed lizards, Pasteurellaceae (*LDA* = 4.85, *p* = 0.0008) at the family level in wild lizards, *Rothia* (*LDA* = 4.65, *p* < 0.0001) at the genus level in frog-fed lizards, *Bacteroides* (*LDA* = 4.59, *p* = 0.03) and *UCG_008* (*LDA* = 4.27, *p* = 0.01) at the genus level in chicken-fed lizards, *Mycoplasma* (*LDA* = 5.07, *p* < 0.0001), *Uruburuella* (*LDA* = 4.79, *p* < 0.0001), and *Paracoccus* (*LDA* = 4.48, *p* = 0.04) at the genus level in egg-fed lizards, and *Capnocytophaga* (*LDA* = 5.50, *p* < 0.0001) at the genus level in wild lizards had a higher proportion (Figure 4). The relative abundance of above bacterial taxa except families Enterobacteriaceae and Pasteurellaceae and genera *Bacteroides*, *Campylobacter*, and *Porphyromonas* differed significantly among animal groups ingesting different items of food (Figure 5).

### The Predicted Metagenomes

**Figure 6:**
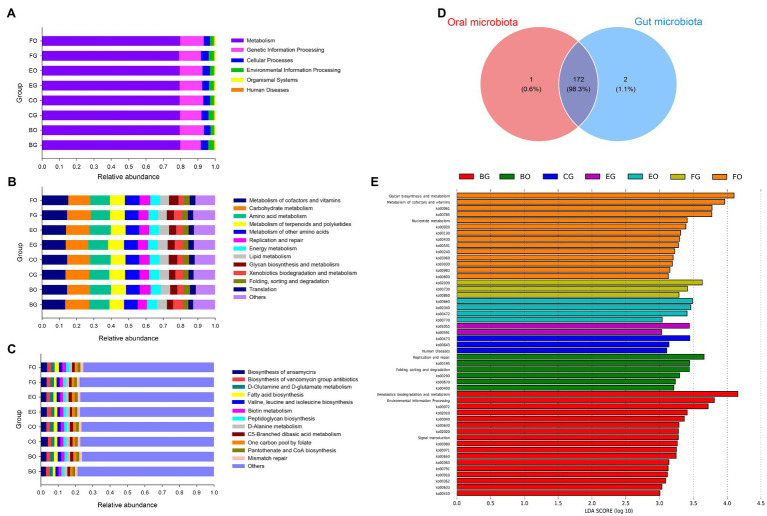
Gene functional categories based on 16S RNA in the gut microbiota at top **(A)**, second **(B)**, and third **(C)** levels of relative abundance, and the Venn diagram of functional gene between the oral and cloacal samples **(D)**. LDA scores reflect the differences in relative abundance among eight groups of microbiota samples **(E)**. Each color in a plot indicates one gene function. Detailed descriptions are shown on the right side of each plot. The colors for others in Plots **B** and **C** indicate all other gene functions not listed in these two plots. See Figure 1 for the definition of every group.

Metabolism held the overwhelming predominance of functional categories at the top level in the oral (79.5 ± 0.2%) and gut (79.6 ± 0.1%) microbiota (Figure 6A). The other three major functional categories at the top level were involved in genetic information processing (12.5 ± 0.3%), cellular processes (4.1 ± 0.1%), and environmental information processing (3.2 ± 0.2%) in the gut microbiota, and genetic information processing (13.6 ± 0.1%), cellular processes (4.0 ± 0.2%), and environmental information processing (2.3 ± 0.1%) in the oral microbiota (Figure 6A). Figure 6B lists the top 12 categories at the second level associated with metabolism and genetic information processing in the oral and cloacal microbial communities. The primary functional categories at the third level were involved in the biosynthesis of ansamycins in both oral and gut microbiota (Figure 6C).

A total of 175 known KO functional genes were identified and the oral and gut microbes shared 172 KEGG functional genes (Figure 6D). LEfSe analysis showed a higher proportion of environmental information processing (*LDA* = 3.81, *p* = 0.02) at the top KEGG level, xenobiotics biodegradation, and metabolism (*LDA* = 4.16, *p* = 0.0001)-related metabolism, as well as signal transduction (*LDA* = 3.28, *p* = 0.0008)-related environmental information processing at the second KEGG level in fed-frog lizards (Figure 6E). Frog-fed lizards had a higher relative abundance in replication and repair (*LDA* = 3.66, *p* = 0.05)-related genetic information processing (Figure 6E). Moreover, glycan biosynthesis and metabolism (*LDA* = 4.10, *p* = 0.02), metabolism of cofactors and vitamins (*LDA* = 3.96, *p* = 0.03), and nucleotide metabolism (*LDA* = 3.41, *p* = 0.003)-related metabolism were enriched in wild lizards (Figure 6E).

## Discussion

Consistent with an earlier study on a diverse array of reptiles including snakes, geckos, and terrapins (Zancolli et al., 2015), Proteobacteria and Bacteroidota were the top two dominant phyla in the oral microbiota of the water monitor lizard (Figure 1). These two phyla also ranked in the top two in the cloacal microbiota of the water monitor lizard (Figure 1). However, positions of the oral (Figure 3B) rather than the gut (Figure 3C) bacterial composition on a two-dimensional plane defined by the first two axes of PCoA differed among animal groups, although the relative abundance of the dominant bacteria in the gut microbiota differed among animal groups (Figures 4, 5). The dominant phyla in the gut microbiota vary among host species. For example, Proteobacteria is one of the top two dominant bacterial phyla in some lizard species (Ren et al., 2016; Tang et al., 2020; Zhou et al., 2020) but not in others (Hong et al., 2011; Kohl et al., 2017; Zhang et al., 2018b). The host-specific gut microbial composition is also documented in fish (Givens et al., 2015) and mammals (Kartzinel et al., 2019).

Diet can rapidly change the gut microbiota composition and thereby affect the host’s body conditions (Clark and Mach, 2016; Jiang et al., 2017; Zhang et al., 2018a). For example, the gut microbial community in loach-fed crocodile lizards (*Shinisaurus crocodilurus*) significantly differs from that in the earthworm-fed and wild conspecifics (Jiang et al., 2017). Food changes accounted for 57% of the total structural changes in gut microbiota, whereas genetic mutation contributed no more than the 12% in mice (Zhang et al., 2010). Interestingly, however, our data showed small differences in the gut microbial relative abundance among animal groups (three groups of captive lizards eating different types of food and one group of wild lizards) and dietary correlates of the oral microbial community, and confirmed earlier findings that the oral microbial composition is host-specific (Zancolli et al., 2015) and physiologically constrained (Graves et al., 2019; Jo et al., 2021).

Our study verified that alpha-diversity of microbiota was affected by diet and that there was a significant difference in alpha-diversity between the oral and gut microbiota. Microbial diversity is affected by many factors including temperature (Bestion et al., 2017), food (Jiang et al., 2017), and age (Videvall et al., 2019). For example, alpha-diversity of the gut bacterial communities generally decreases as temperature increases in the common lizard *Zootoca vivipara* (Bestion et al., 2017) and is significantly higher in herbivorous mammals than in carnivores (Ley et al., 2008). Alpha diversity of the gut bacterial communities increases along with the growth of the age in ostriches (Videvall et al., 2019). In crocodile lizards, captivity increases the gut microbial richness compared with wild conspecifics, presumably because of the complex integration of simple food resources or human contact in captivity (Tang et al., 2020). In this study, bacterial diversity was significantly higher in the gut than in the oral cavity, presumably because the cloacal communities are more variable than the oral ones.

Comprehensive analysis of LEfSe and Mann-Whitney *U* tests showed numerous bacteria with high relative abundance in captive lizards, such as those of the order Pseudomonadales in the oral cavity of egg-fed lizards and the families Dysgonomonadaceae and Comamonadaceae in the gut of egg- and frog-fed lizards (Figures 4, 5). The functions of most of these bacteria are poorly known, but those of the family Dysgonomonadaceae and the genera *Fusobacterium* (Brennan and Garrett, 2019) and *Mycoplasma* (Baseman and Tully, 1997) have been proved to be associated with human diseases (Bridges and Gage, 2021). Bacteria of the genus *Rothia* are commonly found in dental plaque in the oral cavity (Welch et al., 2016) and are associated with heart disease (Pechal et al., 2018) and infectious endocarditis in humans (Boudewijns et al., 2003). Therefore, lizards in captivity may have increased opportunities for transmission of microbiota from human keepers due to frequent contact. In addition, Comamonadaceae at the family level in the gut in the oral cavity had a higher proportion in frog-fed lizards (Figures 4, 5). Comamonadaceae is ubiquitously found in freshwater habitats (Moon et al., 2018), and its enrichment in the gut might be associated with the intake of bullfrogs. This phenomenon might suggest that microbial community obtained from aquatic products could help water monitor lizards better adapt to the environment.

The dominant functional categories of metabolism at the top level in the oral and gut microbiota have been studied in a wide range of reptile taxa. A high proportion of gene function related to metabolism at the top level has been detected in lizards (Tang et al., 2020), snakes (Tang et al., 2019), and turtles (Qu et al., 2020). In the present study, lizards of different groups shared most gene functions, with only a higher proportion of gene function related to environmental information processing and metabolism found in frog-fed lizards (Figure 6). Frog-fed lizards also had a higher relative abundance in gene function-related genetic information processing (Figure 6). Moreover, gene function-related metabolism was enriched in wild lizards (Figure 6). The gene functional differences between frog-fed and wild lizards might be correlated with the abundance of microorganisms in the wild and water environments.

## Conclusion

Proteobacteria and Bacteroidota were the prominent phyla in the oral and gut microbes in the water monitor lizard. The composition of the oral microbiota and the relative abundance of oral and gut microbes were affected by items of food ingested by lizards. Alpha diversity was higher in the gut microbiota than in the oral microbiota. Community richness of oral microbiota was higher in captive lizards than in wide lizards. Inconsistent with previous studies, dietary correlates of the composition of the gut microbiota were not evident in the water monitor lizard. Frog-fed lizards and wild lizards had a higher function proportion-related genetic information processing and metabolism than lizards of other two groups. This difference might result from the high relative abundance of microorganisms in the more complex environments in which frog-fed lizards and wild lizards live. In view of the fact that some identified bacteria have been proved to be associated with human diseases, our study provides an inference that feeding on frogs and aquatic products and reducing human exposure help water monitor lizards maintain a microbiota similar to that in the wild environment.

## Data Availability Statement

All 16S rRNA gene sequences obtained in this study have been deposited in the National Genomics Data Center (NGDC) GSA database (accession number CRA004563).

## Ethics Statement

The animal study was reviewed and approved by The Animal Research Ethics Committee of Nanjing Normal University (Permit No. IACUC 20200511).

## Author Contributions

YD, Y-FQ, and XJ conceived and designed the research. XJ supervised the study. YD, J-QC, QL, J-CF, C-XL, L-HL, HL, and Y-FQ conducted the experiments and collected the data. YD, J-QC, QL, and Y-FQ analyzed the data. Y-FQ and XJ wrote the paper. All authors contributed to the article and approved the submitted version.

## Funding

The study was supported by grants from the Technology Project of Hainan Province (ZDYF2018219), the Hainan Key Program of Science and Technology (ZDXM20110008), the Hainan Key Laboratory of Herpetological Research (HKLHR202002), and the National Natural Science Foundation of China (NSFC) to Y-FQ (Grant no. 31770443 and 32171498).

## Conflict of Interest

The authors declare that the research was conducted in the absence of any commercial or financial relationships that could be construed as a potential conflict of interest.

## Publisher’s Note

All claims expressed in this article are solely those of the authors and do not necessarily represent those of their affiliated organizations, or those of the publisher, the editors and the reviewers. Any product that may be evaluated in this article, or claim that may be made by its manufacturer, is not guaranteed or endorsed by the publisher.
