# Supplementary material for: Dietary Correlates of Oral and Gut Microbiota in the Water Monitor Lizard, Varanus salvator (Laurenti, 1768)
Source: Front Microbiol. 2022 Jan 6;12:771527. doi: 10.3389/fmicb.2021.771527 (PMC8770915; doi:10.3389/fmicb.2021.771527)

Supplementary Table 1 The number of valid reads of each bacterial sample of the oral cavity and gut for Varanids treated with DADA2 and their number of biosamples in the National Genomics Data Center (NGDC) GSA database (accession number CRA004563)

| Individual ID | Sample ID | Group | Reads numbers | Average sequence length | Minimum sequence length | Maximum sequence length | Biosample | Bioproject |
| --- | --- | --- | --- | --- | --- | --- | --- | --- |
| 1 | BG1 | B | 78672 | 422 | 436 | 233 | SAMC433367 | PRJCA005866 |
|  | BO1 | B | 69478 | 423 | 436 | 238 | SAMC433374 | PRJCA005866 |
| 2 | BG2 | B | 46032 | 421 | 436 | 231 | SAMC433368 | PRJCA005866 |
|  | BO2 | B | 70295 | 419 | 431 | 249 | SAMC433375 | PRJCA005866 |
| 3 | BG3 | B | 54339 | 417 | 436 | 232 | SAMC433369 | PRJCA005866 |
|  | BO3 | B | 76288 | 422 | 436 | 231 | SAMC433376 | PRJCA005866 |
| 4 | BG4 | B | 56028 | 420 | 436 | 233 | SAMC433370 | PRJCA005866 |
|  | BO4 | B | 64800 | 421 | 436 | 336 | SAMC433377 | PRJCA005866 |
| 5 | BG5 | B | 55728 | 424 | 431 | 227 | SAMC433371 | PRJCA005866 |
|  | BO5 | B | 69552 | 424 | 431 | 272 | SAMC433378 | PRJCA005866 |
| 6 | BG6 | B | 45148 | 423 | 436 | 230 | SAMC433372 | PRJCA005866 |
|  | BO6 | B | 63331 | 426 | 436 | 238 | SAMC433379 | PRJCA005866 |
| 7 | BG7 | B | 66646 | 420 | 432 | 234 | SAMC433373 | PRJCA005866 |
|  | BO7 | B | 55557 | 424 | 436 | 231 | SAMC433380 | PRJCA005866 |
| 8 | CG1 | C | 62685 | 422 | 436 | 235 | SAMC433381 | PRJCA005866 |
|  | CO1 | C | 69219 | 424 | 436 | 232 | SAMC433389 | PRJCA005866 |
| 9 | CG2 | C | 76747 | 415 | 436 | 238 | SAMC433382 | PRJCA005866 |
|  | CO2 | C | 71176 | 422 | 436 | 238 | SAMC433390 | PRJCA005866 |
| 10 | CG3 | C | 70875 | 426 | 436 | 238 | SAMC433383 | PRJCA005866 |
|  | CO3 | C | 58146 | 424 | 436 | 230 | SAMC433391 | PRJCA005866 |
| 11 | CG4 | C | 68126 | 423 | 436 | 227 | SAMC433384 | PRJCA005866 |
|  | CO4 | C | 64100 | 427 | 436 | 235 | SAMC433392 | PRJCA005866 |
|  |  |  |  |  |  |  | …to be continued | |
| Supplementary Table 1 (Continued) | | |  |  |  |  |  |  |
| Individual ID | Sample ID | Group | Reads numbers | Average sequence length | Minimum sequence length | Maximum sequence length | Biosample | Bioproject |
| 12 | CG5 | C | 70520 | 425 | 436 | 238 | SAMC433385 | PRJCA005866 |
|  | CO5 | C | 50661 | 420 | 437 | 230 | SAMC433393 | PRJCA005866 |
| 13 | CG6 | C | 64207 | 425 | 436 | 238 | SAMC433386 | PRJCA005866 |
|  | CO6 | C | 34551 | 425 | 436 | 238 | SAMC433394 | PRJCA005866 |
| 14 | CG7 | C | 78307 | 418 | 431 | 234 | SAMC433387 | PRJCA005866 |
|  | CO7 | C | 58742 | 425 | 437 | 232 | SAMC433395 | PRJCA005866 |
| 15 | CG8 | C | 84868 | 418 | 432 | 257 | SAMC433388 | PRJCA005866 |
|  | CO8 | C | 56991 | 421 | 436 | 230 | SAMC433396 | PRJCA005866 |
| 16 | EG1 | E | 51504 | 419 | 436 | 232 | SAMC433397 | PRJCA005866 |
|  | EO1 | E | 70375 | 419 | 436 | 237 | SAMC433404 | PRJCA005866 |
| 17 | EG2 | E | 53356 | 417 | 436 | 233 | SAMC433398 | PRJCA005866 |
|  | EO2 | E | 67777 | 425 | 436 | 271 | SAMC433405 | PRJCA005866 |
| 18 | EG3 | E | 49387 | 416 | 436 | 231 | SAMC433399 | PRJCA005866 |
|  | EO3 | E | 87740 | 423 | 431 | 246 | SAMC433406 | PRJCA005866 |
| 19 | EG4 | E | 72114 | 426 | 436 | 238 | SAMC433400 | PRJCA005866 |
|  | EO4 | E | 67745 | 424 | 437 | 251 | SAMC433407 | PRJCA005866 |
| 20 | EG5 | E | 64765 | 415 | 436 | 235 | SAMC433401 | PRJCA005866 |
|  | EO5 | E | 60487 | 421 | 436 | 231 | SAMC433408 | PRJCA005866 |
| 21 | EG6 | E | 68497 | 424 | 436 | 238 | SAMC433402 | PRJCA005866 |
|  | EO6 | E | 60553 | 425 | 436 | 237 | SAMC433409 | PRJCA005866 |
| 22 | EG7 | E | 77833 | 417 | 432 | 238 | SAMC433403 | PRJCA005866 |
|  | EO7 | E | 62743 | 425 | 436 | 235 | SAMC433410 | PRJCA005866 |
| 23 | FG1 | F | 62710 | 416 | 431 | 235 | SAMC433411 | PRJCA005866 |
|  | FO1 | F | 61380 | 423 | 436 | 232 | SAMC433414 | PRJCA005866 |
|  |  |  |  |  |  |  | …to be continued | |
|  |  |  |  |  |  |  |  |  |
| Supplementary Table 1 (Continued) | | |  |  |  |  |  |  |
| Individual ID | Sample ID | Group | Reads numbers | Average sequence length | Minimum sequence length | Maximum sequence length | Biosample | Bioproject |
| 24 | FG2 | F | 68502 | 427 | 436 | 238 | SAMC433412 | PRJCA005866 |
|  | FO2 | F | 52704 | 426 | 436 | 232 | SAMC433415 | PRJCA005866 |
| 25 | FG3 | F | 71235 | 420 | 436 | 238 | SAMC433413 | PRJCA005866 |
|  | FO3 | F | 44487 | 425 | 436 | 231 | SAMC433416 | PRJCA005866 |

Supplementary Table 2 The number of amplicon sequence variants (ASVs) and different bacterial taxonomic units of each oral and gut sample in varanids.

| Individual ID | Sample ID | Group | ASVs | Genus | Family | Class | Order | Phylum |
| --- | --- | --- | --- | --- | --- | --- | --- | --- |
| 1 | BG1 | B | 314 | 144 | 86 | 24 | 56 | 13 |
|  | BO1 | B | 183 | 102 | 65 | 16 | 41 | 11 |
| 2 | BG2 | B | 265 | 130 | 77 | 21 | 47 | 13 |
|  | BO2 | B | 233 | 132 | 86 | 27 | 55 | 16 |
| 3 | BG3 | B | 332 | 169 | 103 | 29 | 63 | 16 |
|  | BO3 | B | 212 | 111 | 70 | 18 | 43 | 13 |
| 4 | BG4 | B | 303 | 153 | 94 | 26 | 59 | 17 |
|  | BO4 | B | 270 | 161 | 104 | 27 | 60 | 18 |
| 5 | BG5 | B | 248 | 132 | 83 | 20 | 53 | 13 |
|  | BO5 | B | 194 | 112 | 69 | 18 | 46 | 13 |
| 6 | BG6 | B | 248 | 131 | 90 | 26 | 58 | 16 |
|  | BO6 | B | 163 | 86 | 62 | 19 | 42 | 15 |
| 7 | BG7 | B | 366 | 186 | 112 | 34 | 71 | 19 |
|  | BO7 | B | 169 | 87 | 60 | 16 | 39 | 11 |
| 8 | CG1 | C | 292 | 150 | 101 | 29 | 64 | 18 |
|  | CO1 | C | 175 | 93 | 67 | 19 | 44 | 13 |
| 9 | CG2 | C | 260 | 127 | 84 | 27 | 53 | 16 |
|  | CO2 | C | 164 | 84 | 60 | 18 | 41 | 12 |
| 10 | CG3 | C | 235 | 124 | 76 | 22 | 46 | 13 |
|  | CO3 | C | 209 | 110 | 74 | 19 | 46 | 14 |
| 11 | CG4 | C | 308 | 152 | 95 | 31 | 62 | 19 |
|  | CO4 | C | 96 | 55 | 43 | 14 | 32 | 9 |
|  |  |  |  |  |  | …to be continued | | |
| Supplementary Table 2 (Continued) | | |  |  |  |  |  |  |
| Individual ID | Sample ID | Group | ASVs | Genus | Family | Class | Order | Phylum |
| 12 | CG5 | C | 221 | 121 | 73 | 18 | 42 | 12 |
|  | CO5 | C | 160 | 88 | 55 | 13 | 37 | 9 |
| 13 | CG6 | C | 225 | 126 | 76 | 21 | 47 | 13 |
|  | CO6 | C | 145 | 77 | 56 | 16 | 34 | 11 |
| 14 | CG7 | C | 202 | 112 | 78 | 24 | 52 | 17 |
|  | CO7 | C | 169 | 82 | 57 | 18 | 35 | 12 |
| 15 | CG8 | C | 331 | 167 | 104 | 30 | 61 | 17 |
|  | CO8 | C | 140 | 92 | 64 | 20 | 44 | 13 |
| 16 | EG1 | E | 194 | 104 | 68 | 17 | 44 | 11 |
|  | EO1 | E | 167 | 89 | 59 | 14 | 35 | 10 |
| 17 | EG2 | E | 258 | 154 | 87 | 24 | 55 | 15 |
|  | EO2 | E | 202 | 117 | 76 | 16 | 43 | 12 |
| 18 | EG3 | E | 233 | 128 | 88 | 28 | 58 | 18 |
|  | EO3 | E | 204 | 113 | 75 | 18 | 45 | 13 |
| 19 | EG4 | E | 281 | 149 | 99 | 32 | 66 | 19 |
|  | EO4 | E | 189 | 99 | 70 | 19 | 43 | 14 |
| 20 | EG5 | E | 300 | 151 | 98 | 26 | 57 | 15 |
|  | EO5 | E | 139 | 75 | 52 | 16 | 38 | 11 |
| 21 | EG6 | E | 334 | 168 | 106 | 36 | 71 | 22 |
|  | EO6 | E | 202 | 113 | 74 | 22 | 49 | 14 |
| 22 | EG7 | E | 317 | 169 | 91 | 26 | 56 | 14 |
|  | EO7 | E | 242 | 129 | 80 | 26 | 52 | 14 |
| 23 | FG1 | F | 198 | 97 | 69 | 18 | 40 | 12 |
|  | FO1 | F | 136 | 73 | 48 | 14 | 29 | 9 |
|  |  |  |  |  |  | …to be continued | | |
| Supplementary Table 2 (Continued) | | |  |  |  |  |  |  |
| Individual ID | Sample ID | Group | ASVs | Genus | Family | Class | Order | Phylum |
| 24 | FG2 | F | 216 | 117 | 75 | 21 | 47 | 13 |
|  | FO2 | F | 117 | 65 | 44 | 11 | 27 | 6 |
| 25 | FG3 | F | 297 | 146 | 91 | 23 | 51 | 13 |
|  | FO3 | F | 138 | 70 | 44 | 13 | 28 | 8 |

Figure S1 Rarefaction curves based on ASVs for every sample. Different color lines represent different samples.


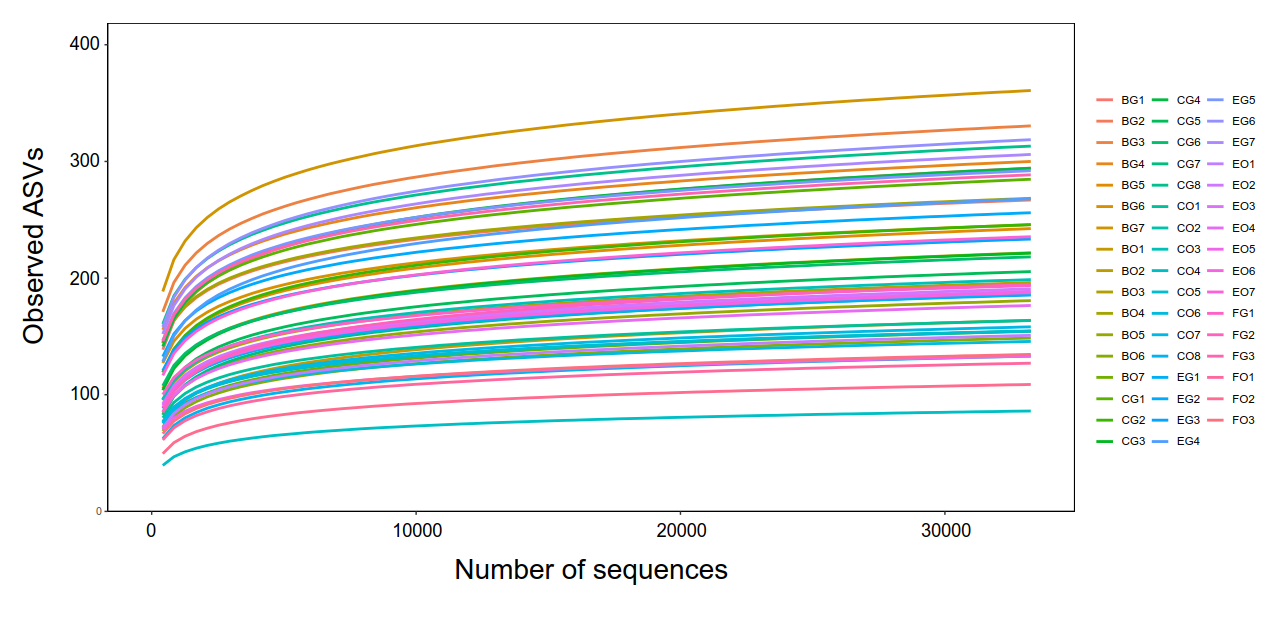

Supplement: Supplementary file 1 [file Table_1.docx]
